# Draft Genome Sequences of Multidrug-Resistant *Acinetobacter* sp. Strains from Colombian Hospitals

**DOI:** 10.1128/genomeA.00868-13

**Published:** 2013-11-27

**Authors:** Emiliano Barreto-Hernández, Laurent Falquet, María T. Reguero, José R. Mantilla, Emilia M. Valenzuela, Elsa González, Alexandra Cepeda, Andrea Escalante

**Affiliations:** Biotechnology Institute, National University of Colombia, Bogotá, Colombiaa; University of Fribourg, Fribourg, Switzerlandb; Swiss Institute of Bioinformatics, Lausanne, Switzerlandc

## Abstract

The draft genome sequences of the strains *Acinetobacter baumannii* 107m, *Acinetobacter nosocomialis* 28F, and *Acinetobacter pittii* 42F, isolated from Colombian hospitals, are reported here. These isolates are causative of nosocomial infections and are classified as multidrug resistant, as they showed resistance to four different antibiotic groups.

## GENOME ANNOUNCEMENT

A major antimicrobial resistance problem in Latin American countries is the growing prevalence of multidrug-resistant (MDR) *Acinetobacter* spp. (1, 2). Four species of *Acinetobacter* have been grouped as the *Acinetobacter calcoaceticus-Acinetobacter baumannii* (ACB) complex because they are genetically similar and difficult to identify with automated or routine laboratory methods (3). Three of these species (*A. baumannii*, *A. nosocomialis*, and *A. pittii*) are associated with nosocomial infections, and in spite of their similarity, they exhibit different resistance profiles (4).

In Colombia, several studies of outbreaks and molecular characterization of *Acinetobacter* spp. have been carried out from approximately 2001 to the present (5–7).

To date, there are no whole-genome sequences of *Acinetobacter* species strains isolated in Colombia available in GenGank/EMBL/DDBJ. This report announces the draft genome sequences of strains *A. baumannii* 107m, *A. nosocomialis* 28F, and *A. pittii* 42F. These multidrug-resistant isolates were selected from an initial set of 139 nosocomial isolates from 20 hospitals in Bogotá collected from 2005 to 2010.

Whole-genome shotgun (WGS) sequencing was performed using the HiSeq 2000 Illumina paired-end platform and the 454 pyrosequencing mate-pair (7.5-kb insert) platform. Sequences were *de novo* assembled using Velvet 1.2.07 (8) and Newbler 2.7 (Roche Diagnostics Corporation) and were mapped using BWA 0.6.1 (9) to the reference genomes of strains *A. baumannii* ATCC 17978, *A. nosocomialis* RUH 2624, and *A. pittii* SH024. Automatic annotation was done through the NCBI Prokaryotic Genomes Automatic Annotation Pipeline (PGAAP) (10) and by the NMPDR RAST 4.0 server (11). Annotations were visualized and manually curated with Artemis 14.0.0 (12).

The *A. baumannii* 107m draft genome has a total of 3,954,000 bp consisting of 56 contigs. There are 3,735 predicted genes, with an average size of 912 bp, 3,796 coding sequences (CDSs), and 57 tRNAs. A total of 2,884 CDSs were assigned to Clusters of Orthologous Groups (COGs), while the CDSs were classified into 441 metabolic subsystems by RAST. The *A. nosocomialis* 28F draft genome has a total of 3,833,431 bp consisting of 92 contigs. There are 3,626 predicted genes, with an average size of 910 bp, 3,680 CDSs, and 54 tRNAs. A total of 2,849 CDSs were assigned to COGs, while CDSs were classified into 433 metabolic subsystems by RAST. Finally, the *A. pittii* 42F draft genome has a total of 3,782,611 bp consisting of 72 contigs. There are 3,611 predicted genes, with an average size of 910 bp, 3,665 CDSs, and 54 tRNAs. A total of 2,832 CDSs were assigned to COGs, while CDSs were classified into 423 metabolic subsystems by RAST. Genes associated with multidrug resistance were annotated and compared with other *Acinetobacter* genomes that had already been sequenced.

### Nucleotide sequence accession numbers.

These whole-genome sequencing projects have been deposited at DDBJ/EMBL/GenBank under the following accession no.: CBSG00000000 (*A. baumannii* 107m), CBSD00000000 (*A. nosocomialis* 28F), and CBRO00000000 (*A. pittii* 42F). The versions described in this paper are CBSG01000000, CBSD01000000, and CBRO01000000.
